# Population size and structure of Grant’s gazelle and lesser kudu in Geralle National Park, Southeastern Ethiopia

**DOI:** 10.7717/peerj.18340

**Published:** 2024-11-15

**Authors:** Melkamu Aychew, Zerihun Girma, Zenebe Ageru Yilma

**Affiliations:** 1 Geralle National Park, Ethiopian Wildlife Conservation Authority, Addis Ababa, Ethiopia; 2 Department of Wildlife & Ecotourism Management, Wondo Genet College of Forestry & Natural Resource, Hawassa University, Hawassa, Sidama, Ethiopia; 3 Natural Resource, Mizan ATVET College, Mizian Teferi, SWERS, Ethiopia

**Keywords:** Abundance, Density, Distance sampling, Habitat, Season, Species

## Abstract

**Background:**

Grant’s gazelle and lesser kudu remain widespread within and outside protected areas. Current pressures on their populations, human encroachment and disturbance associated habitat modifications, and excessive grazing pose further threats to the species. The estimation of density and abundance of species has significant value for sustainable wildlife management in Geralle National Park (GNP) and also contributes towards a more accurate global population estimate.

**Result:**

Using distance sampling methods, the lowest Akaike Information Criterion (AIC) (close to zero) and Chi-square tests (*P* value > 0.05) showed that the hazard rate key function with an un-equal interval group model was selected for estimation of density and abundance. The density of species was 1.7 ± 0.5, 2.07 ± 0.7, gazelle/km^2^ and 1.39 ± 0.3, 1.92 ± 0.42, lesser kudu/km^2^ during the dry and wet seasons, respectively. Population density, abundance, and structure showed habitat and seasonal differences in observation. More individuals of both species were recorded during the wet season as compared to the dry season. Grassland was favored by Grant’s gazelle, while lesser kudu preferred woodlands. Both species exhibited a female-biased sex ratio, indicating potential for future population growth prospects.

**Conclusion:**

It can be concluded that GNP is home to viable populations of Grant’s gazelle and lesser kudu, and season has influenced population abundance and distribution due to resource availability variations among seasons. The female-biased sex ratio indicates the future population growth prospects for the two species.

## Introduction

African herbivores, such as gazelles and kudus, play crucial roles in the functioning of Africa’s grassland and woodland ecosystems. They influence the vegetation dynamics of the ecosystem, serve as prey population for charismatic carnivores, and contribute to income generation through eco-tourism. However, through continued anthropogenic pressures, their population is declining, and there is an urge for immediate conservation attention (Okello et al., 2016).

Gazelles (Bovidae, Mammalia) generally inhabit the dry, open savannah grasslands and scrub habitats of Africa (Kingdon, 2015). Grant’s gazelle (*Nanger granti*) is one of the members of the genus Gazella (Nowak, 1999). Grant’s gazelles are distributed in Sudan, Ethiopia, Kenya, and Tanzania (Nowak, 1999; Kingdon, 2015). The largest surviving populations occur in Ethiopia (Omo-Mago-Murule-Chew Bahir and Borana), Kenya (the Northern rangelands, Kajiado, Mara, Tsavo and Laikipia) and Tanzania (Serengeti) (IUCN, 2024).

Groves & Grubb (2011) and Kingdon (2015) believed that there are two sub-species of lesser kudu: the Northern lesser kudu, *Tragelaphus imberbis imberbis*, and the Southern lesser kudu, *Tragelaphus imberbis australis*, although it is yet to be confirmed by genetic analysis. The Northern lesser kudu is said to occur in the lowlands of east and central Ethiopia (the awash area) and the Northwest Somalia (IUCN/SSC-ASG, 2008; Kingdon, 2015). The Southern lesser kudu live in the lowlands of southern Ethiopia, Somalia, the extreme south-east of Sudan, the extreme northeast of Uganda, north, central, and southern Kenya, and eastern Tanzania (Kingdon, 2015). Lesser kudu occurs in the forest and bushlands of Ethiopia throughout most of its former range in the eastern and southern lowlands and is found in protected areas of Ethiopia, such as Mago and Geralle National Parks, Chew Bahar, Tama and Alledeghi Wildlife Reserves, Babille, and Yabelo Wildlife Sanctuaries, Borana, and Murule Controlled Hunting Areas.

Both Grant’s gazelle and lesser kudu population trends are declining in most of their ranges. Current pressures on their populations including human encroachment and disturbance, as well as associated habitat modifications and excessive grazing, pose further threats to these species (Okello et al., 2016). Most of the populations of both species occur in protected areas (PAs) where there is better protection and conservation effort (Charie, 2004; Estes, 2012). In Ethiopia, populations of Grant’s gazelle and lesser kudu in the wild, in most of its ranges, have suffered decline due to habitat degradation and poaching (Charie, 2004; Stuart & Stuart, 2006). Population densities of the two species are determined by season and habitat quality. It has been reported that (Admasu, Bekele & Asefa, 2016; Alemu, Mundanthurai & Bekele, 2016) abundance and density of species is affected by the availability of food and cover, which is influenced mainly by vegetation composition and structure. The abundance and availability of food and cover of large herbivores is influenced by season (Odadi et al., 2011; Admasu, Bekele & Asefa, 2016; Alemu, Mundanthurai & Bekele, 2016). The main food sources of large herbivores such as grazing grasses and browsing leaves availability and quality varies between seasons (Thapa et al., 2021). Rainfall availability and abundance affect grass and browse nutritional quality and digestibility (Ahrestani et al., 2016). During the dry season, due to limitations in rainfall availability vegetation cover becomes sparser and food and water availability becomes limited (Matsuo et al., 2024; Waltert, Meyer & Kiffner, 2009). Since grasses and leaves dry out during dry season because of limited rain, the availability of cover and foods available for large herbivores declines (Ryan et al., 2016) During the dry season as opposed to the wet season, grasses in general are more fibrous, *i.e.,* they have a greater proportion of cell wall (cellulose and hemicellulose) to cell content, and have higher levels of abrasive components like silica (Kaiser et al., 2009), which reduce the nutritive quality of the foraging grass. The availability of resources within different habitat types may influence the time spent in each habitat type. Herbivores’ density and abundance vary among the availability and quality of habitat resources such as cover, food, water and space (Boyce et al., 2016). In heterogeneous environments, the availability and distribution of different habitats, as well as the quality and availability of habitat resources determine large herbivore abundance and density (Avgar, Betini & Fryxell, 2020). In most habitats, plant communities determine the physical structure of the environment, and therefore, have a considerable influence on the distributions and interactions of animal species (Avgar, Betini & Fryxell, 2020). Plant structure among habitat types, determines the quality and quantity of cover and digestible material available to herbivores (Hopcraft, Olff & Sinclair, 2010). In general, the habitat resources availability and quality variations between seasons and habitat types determine large herbivores abundance and density. Therefore, our understanding of these interactions will help determine those environmental features that guarantee or predicts fitness and survival of wildlife species in given spatial and temporal scales.

Due to a lack of exact population estimates over its ranges, Grant’s gazelle and lesser kudu global population estimates are not certain. Over some ranges of species in Ethiopia, few studies attempted to estimate the size of local populations of Grant’s gazelle and lesser kudu. For instance, a study conducted by Alemu, Mundanthurai & Bekele (2016) provided an estimation of the population of Grant’s gazelle in Nech Sar National Park. Similarly, the population size of lesser kudu in Tululujia Wildlife Reserve, Southwestern Ethiopia, was estimated by Belete & Melese (2016). However, the population status of the two species in most of the localities is not known due to a lack of population studies in most of the species’ localities. Furthermore, the rainfall availability and amount that determine habitat resources vary among localities. The habitat resource variations among localities influence population structure of Grant’s gazelle and lesser kudu, urging the need for exploring new populations of the species elsewhere. The increasing human population around Geralle National Park and the consequent expansion of settlement, and grazing are threatening the survival of species and their habitats (Asefa, Mengesha & Aychew, 2017). The population structure of the two species is also not known because of lack of local population studies. Due to this, it is not clearly known whether or not there exists healthy population that determines the prospects of the populations of the two species in the future. Meanwhile, few studies elsewhere in Ethiopia have reported population decline across most of its historic ranges, mainly due to habitat modifications and poaching (Asefa, Mengesha & Aychew, 2017). This makes the population status of the two species remain largely unknown in Ethiopia, despite the ongoing threats to their habitats.

The dearth of population studies on the two species, *N. granti* and *T. imberbis imberbis*, and possible population structure variations among season and habitat types in various localities, accentuates the necessity for exhaustive population studies of these species in various localities. This study is conceived to enrich our comprehension of the population estimate and structure, with a particular focus on the abundance of the species within a specific area or habitat, age, and sex distributions. Such insights are instrumental in formulating efficacious management strategies that aim at conserving these species and their habitats. In a nutshell, information on population estimates and structure has vital benefits as it enables managers to make effective conservation management decisions.

Consequently, the study seeks to look at possible seasonal and habitat variations in the size and composition of the lesser kudu and Grant’s gazelle populations in the context of the study on the population dynamics of these species in Geralle National Park. It specifically hypothesizes that, in Geralle National Park, there is (i) seasonal variation in density and abundance, (ii) habitat influences density and abundance, and (iii) how both habitat and season play out across different feeding guilds of herbivores.

Therefore, the study aimed at determining the population size and structure of *N. granti* and *T. imberbis* within the Geralle National Park (GNP), thereby contributing to the broader conservation efforts for these species. This endeavor is expected to provide valuable insights into the differences in population of these species across various habitat types and during two distinct seasons, which is crucial for their long-term survival and conservation.

## Materials & Methods

### Description of the study area

**Figure 1:**
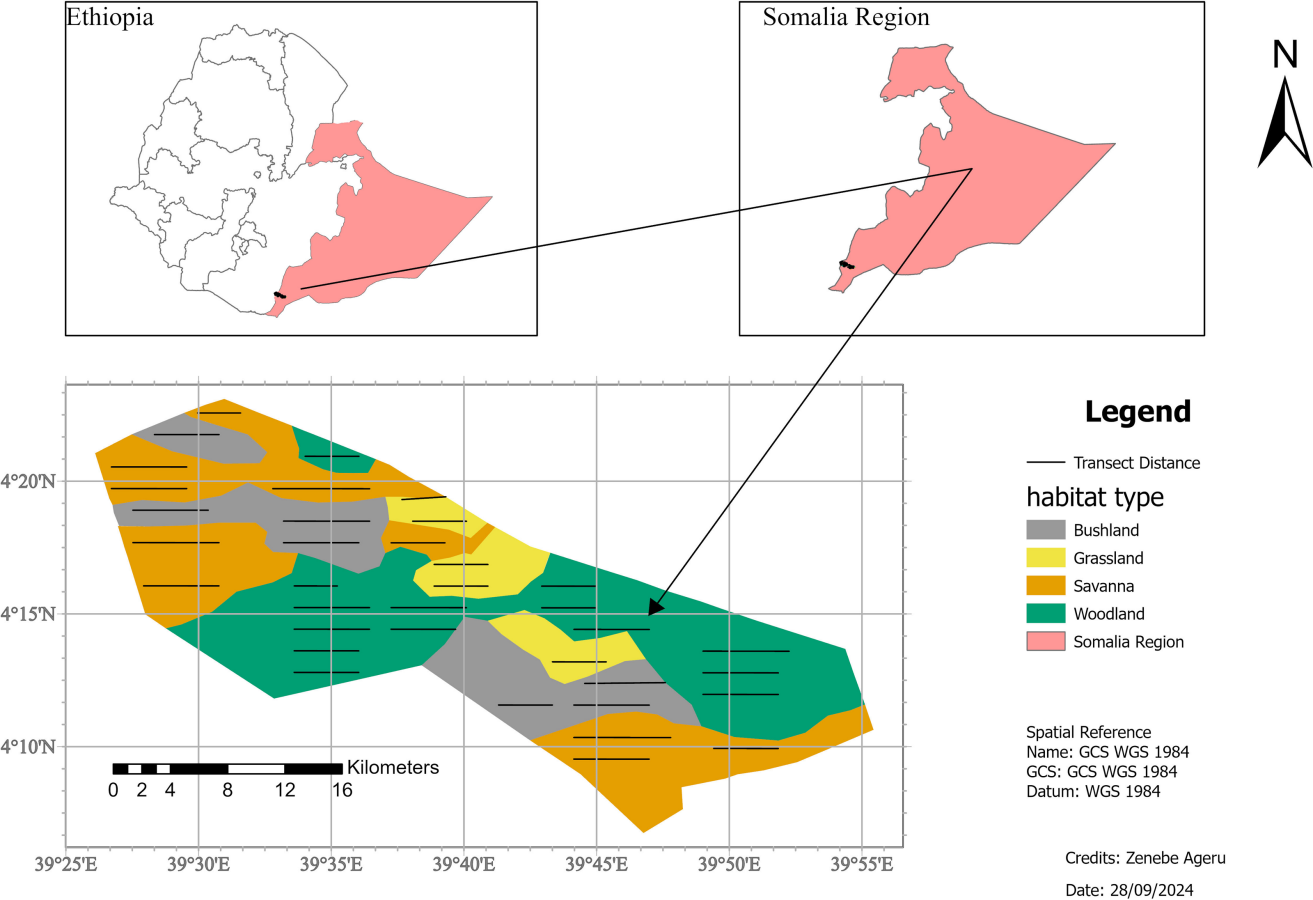
Location map of the study area, Geralle National Park South eastern Ethiopia (Melkamu Aychew, 2021). Map source: https://gadm.org/maps/ETH.html.

Geralle National Park (GNP), which was established in 2006 (Asefa, Mengesha & Aychew, 2017), is located in the south-eastern part of Ethiopia in the Somali National Regional State (SNRS) in Dawa administrative zone, Hudet woreda (district) (Fig. 1). The National Park is bounded to the north by the Liban woreda of the Guji zone (Oromia Region), to the west by the Arero woreda of Borena zone (Oromia Region), to the east by Mubarak Woreda of Dawa zone (SNRS), and southeast by Moyale woreda of the Dawa zone (SNRS). The park is located 709 km from Addis Ababa *via* the Nagelle Borena highway and 901 km *via* Moyale. The park is divided into west and east parts. The west part covers around 692 km^2^ and the east part covers 1,042 km^2^ including the adjacent area of the Dawa ecosystem (Heaven, 2014). West GNP, where the present study was conducted, is situated between 4^∘^0′0″ to 4^∘^30′0″N latitude and 39^∘^30′ to 39^∘^50′E longitude (Fig. 1).

The topography of the National Park is mostly characterized by low land and plains. The altitude ranges as low as 800 m above sea level on the banks of the Dawa River and as high as 1,380 m above sea level on top of the escarpments (Asefa, Mengesha & Aychew, 2017). The rainfall regime in GNP is bimodal, with a long rainy season between March and May with a peak in April, and a short rainy season between September and November, with a peak in October. The mean annual rainfall for the period 2005–2018 was 594.9 mm (NMA, 2021). The peak mean monthly rainfall was in April (132.5 mm) and October (121.0 mm). The least mean monthly rainfall was in January (8.9 mm) (NMA, 2021). The hottest months are January and February, and temperatures fluctuate between 28.3 °C and 29.1 °C. The mean annual minimum temperature was 12 °C. The mean monthly maximum temperature was 29.1 °C., while the minimum was 23.9 °C (NMA, 2021).

The most dominant habitat types in the park are: grassland, woodland, wooded grassland, and open bushland (Asefa, Mengesha & Aychew, 2017). The dominant vegetation type is *Acacia-comiphora*, *Balanites aegyptiaca*, *Boswellia spp*, *Acacia mellifera*, *Acacia brevispica*, *Acacia oerfata*, and *Grewia spp* are the dominant tree species in the park (Asefa, Mengesha & Aychew, 2017). About 42 mammalian species were recorded from the park (Heaven, 2014). Some of the most common mammals include, Beisa Oryx (*Oryx beisa*), Grant’s gazelle (*Nanger granti*), Gerenuk (*Litocranius walleri*), lesser kudu (*Tragelaphus imberbis*), cheetah (*Acinonyx jubatus*), lion (*Panthera leo*) and leopard (*Panthera pardus*). Moreover, endangered and critically endangered species such as African Elephant (*Loxodonta africana*), and African wild dog (*Lycaon pictus*) inhabit the national park. Two hundred species of birds have been identified in the park (Alemu, 2004; Heaven, 2014). Increasing human population around Geralle National Park and consequent expansion of settlement, and grazing are threatening the survival of species and their habitats (Asefa, Mengesha & Aychew, 2017).

### Methods

### Data collection

**Figure 2:**
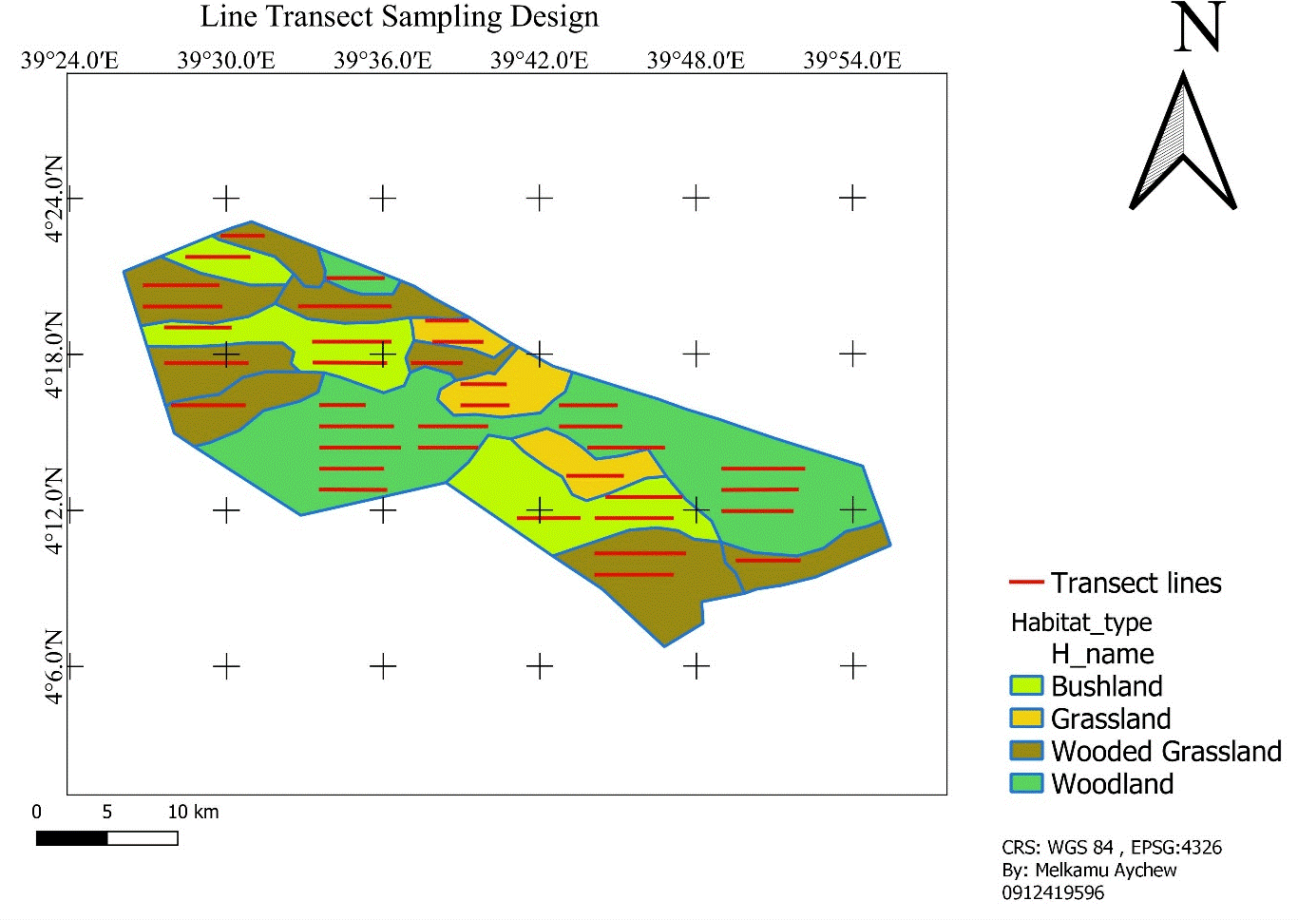
Sampling line transect layout. Map source: https://gadm.org/maps/ETH.html.

We laid transects according to the available habitat types namely; grassland, bushland, wooded grassland, and woodland. Transect lines were established based on the stratified habitat types in a systematic random sampling design and proportional to the size of each habitat type to minimize sampling bias and achieve representativeness. Accordingly, there were a total of 36 transect lines: five (5) in grassland, seven (7) in bushland, ten (10) in wooded-grassland, and fourteen (14) in woodland (Fig. 2). A transect line varied in length from 2.3 km to 6.8 km (totally 165.4 km transect line distance was determined). Adjacent transects were placed with a minimum of 1,500 m apart based on vegetation type, and transect lines were roughly parallel to each other and their ends were at least 500 m from the habitat edges. The 1500 m of space left between consecutive transects was used to avoid double counting. QGIS was used to plot selected transects using systematic random sampling onto the study area map (Fig. 2; QGIS Development Team, 2024). The same transects were used for surveys that were conducted during the wet and dry seasons. This method enabled an all-seasons survey that was uniform across all habitats.

Data collection was conducted during both the wet and dry seasons. The wet season data was collected from April to May in 2020, while the dry season data was collected from August to September in 2021. Surveys were carried out during the early morning hours (from 06:30 to 10:30 a.m.) and late in the afternoon (from 03:00 to 6:30 p.m.), when most mammals are believed to be active (Buckland et al., 2009). This schedule was maintained to ensure the accuracy and relevance of the data collected. We used Global Positioning System (GPS) (Garmin 60/78, Oregon, USA) and handheld bearing compass (Suunto KB-14/360RG, Vantaa, Finland) to navigate to each line transect by walking at a constant speed of 1.25 km/hr (Peres, 1999). The data collection was made through direct observations with the naked eye and aided by a 7 ×35 binoculars. A motorbike was used in this survey by riding slowly at speed of 20–30 km/hr (Keeping & Pelletier, 2014) in most habitat types and transects. A motorbike survey was used due to the plain topography and open habitats. Grassland, wooded grassland, and woodlands are relatively open and have more or less plain topography, so motorbike was used. However, out of seven transects in bushland habitat three transects were covered by thick bushes and it was not possible to transverse the transects using motorbike, hence those transects were walked on foot. Since transects were surveyed predominantly using motorbike, there were not as many variations derived from sampling method. Hence, the cumulative effect on animal behavior and detection functions remain more or less constant. Binoculars were used for proper sex and age identification. The individuals seen within less than 50 m of the nearby group were recorded as members of the same group (Hillman, 1987).

A total of six (6) people (a minimum of seven years of experienced scouts and degree-holder experts in wildlife and related disciplines) and a researcher were involved during the survey and transects nearby with no clear geographic boundaries were surveyed simultaneously. Two days of data collection training were provided for field assistants on how to operate the GPS, count animals, identify sex and age, and record data on datasheets. Per transect, three experienced people were assigned to each transect in the survey. The start and end of the coordinates of each transect were saved in GPS to ensure transects were not repeated during each counting session. Double counting of the same individual herd was avoided using easily recognizable features of an individual’s herd size (total numbers in the herd), age, distinct body deformations, cut tail and ear, and composition (Wilson & Delahay, 2001).

The process of determining age and sex in lesser kudu and Grant’s gazelles was conducted using a variety of physical characteristics. For the lesser kudu, physical characteristics such as body size and the presence or absence of horns were considered (notably, the female lesser kudu, or ewe, is smaller and lacks horns compared to the male, or ram). Both sexes have white markings on the throat and inner legs. In the case of Grant’s gazelles, similar physical characteristics were used for age and sex determination. The body size of Grant’s gazelles can provide an initial clue, with males generally being larger than females. Both sexes carry long, slender horns (Stuart & Stuart, 2006). Morphological development, coloration, and structural organs like the tail, and ears were used to determine the approximate age (Sutherland, 2006). Following the categorization proposed by Lewis & Wilson (1979), the age and sex composition of the species were recorded. This classification system comprises three primary age groups: juveniles, sub-adults, and adults. The juveniles represent the youngest group, while the sub-adults serve as an intermediate group. The adult category encompasses mature individuals. During the census, each individual was sexed as either male or female, and their age was assessed in line with above three age categories. However, it is noteworthy that the sex determination of juveniles and calves posed a significant challenge due to the difficulty in identifying their genital organs. This complexity often results in these groups being categorized without a specific sex assignment.

### Data analysis

The data was organized into a data frame, encompassing variables such as stratum, area, perpendicular distance, transect length, cluster size, species, session, and season. The data manipulation and cleaning were performed using R software version 4.3.3 (R Core Team, 2024). For the analysis of population size, density, and model selection procedures, the distance sampling software version 7.5 (Thomas et al., 2010) supplemented by the MRDS package in R software 4.3.3 was used (Laake et al., 2023; Burt et al., 2014). Variance estimation was grounded on the principles outlined by (Buckland et al., 2001) employing the delta method with empirical variance. Each habitat type was analyzed as a separate stratum, and each species and season were analyzed using a data filtering method in a distance software analysis window (Thomas et al., 2010). Population density for each species was calculated as follows:

(1) \begin{eqnarray*}\hat {D}= \frac{n}{2wL{\hat {P}}_{a}} \end{eqnarray*}

(Buckland et al., 2015; Buckland et al., 2001; Buckland et al., 1993) where, ${\hat {P}}_{a}$ = probability of detection, n number of observations, w = width of the transect, and L = transect distance. 0 < Pa < 1. Pa = *μ*/w where *μ* = $\int \nolimits _{0}^{w}(gx)$)dx. assume that animals on the line are certain to be detected at g(0) = 1

Conventional distance sampling (CDS) is a widely used methodology for estimating the density or abundance of a population. Specifically, distance sampling method major outputs such as (i) detection probability, (ii) population density, and (iii) model selection criteria were used. For determining the detection probability of the two species the Multiple-observer Distance Sampling (MRDS) analysis engine were used. The MRDS engine is an R package for use primarily with double-platform line transect data, where the assumption of certain detection on the line can be relaxed (Laake & Borchers, 2004). Double-platform methods are widely used in both aerial and ground survey of mammals (Borchers & Burnham, 2004), but are potentially useful in many situations where objects at zero distance are difficult to detect. The MRDS analysis engine, which operates on a single observer model, was utilized with a constant parameter from the CDS engine. Despite the MRDS single observer model not introducing additional features compared to the CDS engine, it generates comprehensive summary estimates at both the global and stratum levels when a set of data records is used. These estimates are considered more insightful and detailed than those produced by the CDS engine (Thomas et al., 2010). Both grouped and un-grouped perpendicular distances were performed during model selection procedures to consider categorical and non-categorical data. The distance model selection procedure was between half-normal and hazard-rate key functions for grouped and ungrouped perpendicular distances in the MRDS engine with constant CDS parameters. The best-fitted model was selected according to Akaike’s Information Criterion (AIC) and Chi-square goodness test. Observation differences among habitat, season, and species were performed using two-way analysis of variance (ANOVA). Type III sum of squares was used to overcome unequal sample size designs. The Poisson regression model, which is recommended for analyzing discrete distributions (count data), was used (Tutz, 2011). In this model, transect distances, which vary in length, were used to find the effective strip width as shown in Eq. (2). This approach was employed to analyze the deviance table (ANOVA). We fitted quasi poisson regression to reduce overdispersion. The optimal Poisson regression model was identified using a stepwise selection process, which simplified the predictor variables in the full model (James et al., 2021).

(2) \begin{eqnarray*}\mathrm{n}={e}^{\alpha +{\beta }_{1}\cdot \text{species}+{\beta }_{2}\cdot \text{season}+{\beta }_{3}\cdot \text{habitat}+\log \nolimits (\text{Transect distances})}\end{eqnarray*}

where *α* represents the intercept, *β* stands for the coefficient, and ‘n’ denotes the number of observations. The factors being considered are habitat type, season, and species. The offset is transect distances.

## Results

### Density and abundance

During the dry and wet seasons 125 and 134 individuals of Grant’s-gazelle (*Nanger granti*) and 100 and 134 individuals of lesser kudu (*Traglaphus imberbis*), respectively, were recorded (Table 1). Species, habitat types and seasonal variations had significant and non-significant associations effect of Poisson regression model. Chi-square test analysis on habitat *vs* species (*χ*^2^ = 397.71,  DF = 3, *P*-value < 0.001) and habitat *vs* season (*χ*^2^ = 8.37 DF = 3, *P*-value = 0.039) showed significant associations. Species *vs* season (*χ*^2^ = 6.80 DF = 3, *P*-value = 0.07) showed an insignificant association. Species (*χ*^2^ = 14.3 DF = 3, *P*-value = 0.0024), habitat (*χ*^2^ = 218.04 DF = 3, *P*-value < 0.0001) and season (*χ*^2^ = 6.31 DF = 1, P-Value = 0.012) show significant associations. The wet season showed a higher record of observation than the dry season (*P*-value < 0.0001). In habitat comparison, there were significantly higher numbers of observations in grassland than in the three habitats (*P*-value <0.0001). bushland and wooded grassland have insignificant records (*P*-value = 0.3418), however, these two habitats showed a higher record than woodland habitat (*P*-value <0.0001). For pair wise multiple comparison, there was higher observation in grassland habitat in the wet than grassland in the dry season (*P*-value < 0.001).

**Table 1:** Grant’s gazelle and lesser kudu population size records per habitat types and seasons.

**Habitat**	**Covered area** **(km** **2** **)**	Number of observation (N)			
		Lesser kudu		Grant’s gazelle	
		Dry	Wet	Dry	Wet
**Bushland**	20.4	40	55	0	3
**Grassland**	9.86	0	0	93	106
**Wooded-grassland**	30.78	23	29	32	25
**Woodland**	38.4	37	50	0	0
**Total**	99.24	100	134	125	134

There were significantly higher population records of Grant’s gazelle in the grassland than other habitat types (*P*-value < 0.001). Lesser kudu population record in bushland was significantly higher than woodland habitat (*P*-value = 0). Lesser kudu exhibited a higher population record than Grant’s gazelle in bushland habitat. Gazelle had a higher population observation record than lesser kudu in grassland habitats, whereas lesser kudu showed higher records in bushland, wooded-grassland, and woodland habitats (Table 1). The minimum population observations were 100 and 125 individuals for lesser kudu and Grant’s gazelle, respectively, during the dry season (Table 1). The maximum number of observations was 134 individuals for both species during the wet season.

The lowest AIC, Δ*AIC* (close to zero) and Chi-square tests (*P*-value > 0.05) showed that the hazard rate key function with un-equal interval group model was selected (Table 2). The *P*-value for the Chi-square tests for all species under two seasons was fitted (*P*-value > 0.05). *P*-value for Grant’s gazelle, and lesser kudu were 0.07 and 0.09 in the dry season and 0.17 and 0.16 in the wet season, respectively. The estimated detection probability (${\hat {P}}_{a}$) (percentage) for Grant’s gazelle varied between seasons; in the wet season the detection probability was ${\hat {P}}_{a}$ = 71 ± 9 Standard Error (SE), while in the dry season it increased to a maximum of ${\hat {P}}_{a}$ = 80 ± 5 (SE). For lesser kudu the detection probability was ${\hat {P}}_{a}$ = 77 ± 5 (SE) in the dry season and ${\hat {P}}_{a}$ = 81 ± 7 (SE) was recorded in the wet season (Table 2). Grant’s gazelle estimates of density and abundance coefficient variation in two habitat types were greater than 30% and less than 65%; however, the overall coefficient variation (CV%) was less than 35% (30%) during the dry season and greater than 35% (36%) during the wet season. Grant’s gazelle occurred in low densities in bushland and woodland habitats. Lesser kudu estimates of density and abundance coefficient variation (CV%) in three habitat types were greater than 25% and less than 45%; however, the overall coefficient variation was less than 30% (22%) for both seasons. The estimated overall total population size and density of each species in each habitat type of the species in the GNP during each season is shown in Table 3 below. The highest density of Grant’s gazelle and lesser kudu were recorded in grassland and bushland habitats, respectively (Table 3). The density of Grant’s gazelle in the grassland habitat during the dry season averaged 11.23 (95% CI [5.06–24.92]-; *p* < 0.001, DF = 10), whereas averaged 15.4 (95% CI [6.31–37.59]; *p* < 0.001, DF = 11) during the wet season (Table 3). The density estimate of lesser in the bushland habitat during the dry season averaged 2.37 (95% CI [1.1–5.11]; *p* < 0.001, DF = 15), whereas averaged 3.43 (95% CI [1.49–7.9]; *p* < 0.001, DF = 14) during the wet season (Table 3).

**Table 2:** Grant’s gazelle and lesser kudu population size records per habitat types and seasons. Model selection for multi-species survey.

**Species**	**Model**	**Dry season**					**Wet**				
		P-v	Pâ	se Pâ	ΔAIC	AIC	P-v	Pâ	se Pâ	ΔAIC	AIC
**Grant’s gazelle**	Hn ungroup	0.04	0.90	0.30	3.48	262	0.01	0.71	0.23	2.85	200
	Hn grouped	0.12	0.72	0.23	3.6	119	0.05	0.61	0.21	0	80
	Hr ungroup	0.02	0.86	0.05	0	259	0.01	0.71	0.09	0	180
	Hr grouped	0.07	0.80	0.05	0	115	0.17	0.71	0.09	3.60	119
**Lesser kudu**	Hn ungroup	0.05	0.79	0.16	1.49	451	0.0	0.78	0.16	8.99	520
	Hn grouped	0.12	0.65	0.12	0	220	0.01	0.68	0.14	8.21	228
	Hr ungroup	0.07	0.83	0.07	0	449	0.01	0.79	0.05	0	511
	Hz grouped	0.09	0.81	0.07	0.17	201	0.16	0.77	0.05	0	220

**Notes.**

NB Hn half-normal key function Hr hazard rate key function Pâ probability of detection se Pâ Standard error for detection probability P-v the *P*-value for the Chi-square tests

**Table 3:** Population and density estimates of Grant’s gazelle and lesser kudu per habitat types and seasons.

Species	Habitat	Dry season								Wet season							
Grant’s Gazelle		N	N se	95% CI	*D*	D se	95% CI	CV	DF	N	N se	95% CI	*D*	D se	95% CI	CV	DF
Bushland	0	0	0-0	0	0	0-0	0	0	30	30.69	5–185	0.21	0.21	0.03–1.26	1.01	13
Grassland	735	269.68	331–1631	11.23	4.12	5.06–24.92	0.37	10	1008	426.15	413–2460	15.4	6.51	6.31–37.59	0.42	11
Wooded grassland	282	140.45	106–754	1.21	0.6	0.45–3.24	0.5	20	265	164.83	81–871	1.14	0.71	0.35–3.74	0.62	21
Woodland	0	0	0-0	0	0	0-0	0	0	0	0	0-0	0	0	0-0	0	0
Total	1017	306.7	0.3–543	1.4	0.42	0.75–2.62	0.3	15	1303	469.59	621–2735	1.8	0.65	0.86–3.77	0.36	16
Lesser kudu	Bushland	348	129	162–748	2.37	0.88	1.1–5.11	0.37	15	503	202.47	219–1157	3.43	1.38	1.49–7.9	0.4	14
	Grassland	0	0	0-0	0	0	0-0	0	0	0	0	0-0	0	0	0-0	0	0
	Wooded grassland	210	73	104–423	0.9	0.31	0.45–1.82	0.35	22	279	107.02	129–604	1.2	0.46	0.55–2.6	0.38	20
	Woodland	328	117	162–664	1.17	0.41	0.58–2.36	0.36	31	466	134.59	262–831	1.66	0.48	0.93–2.95	0.29	30
	Total	886	200	568–1383	1.22	0.27	0.78–1.9	0.23	58	1248	273.17	806–1933	1.72	0.38	1.11–2.66	0.22	39

**Notes.**

N.B estimate of density mammals (number/km^2^) N estimate of number of mammals in specified area Se standard error CV coefficient of variation CI Confidence Interval DF Degree of Freedom

**Figure 3:**
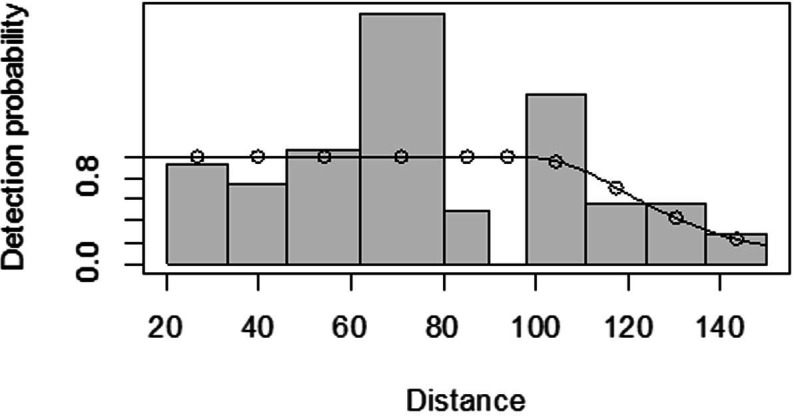
Grant’s gazelle species detection function plot during dry season.

**Figure 4:**
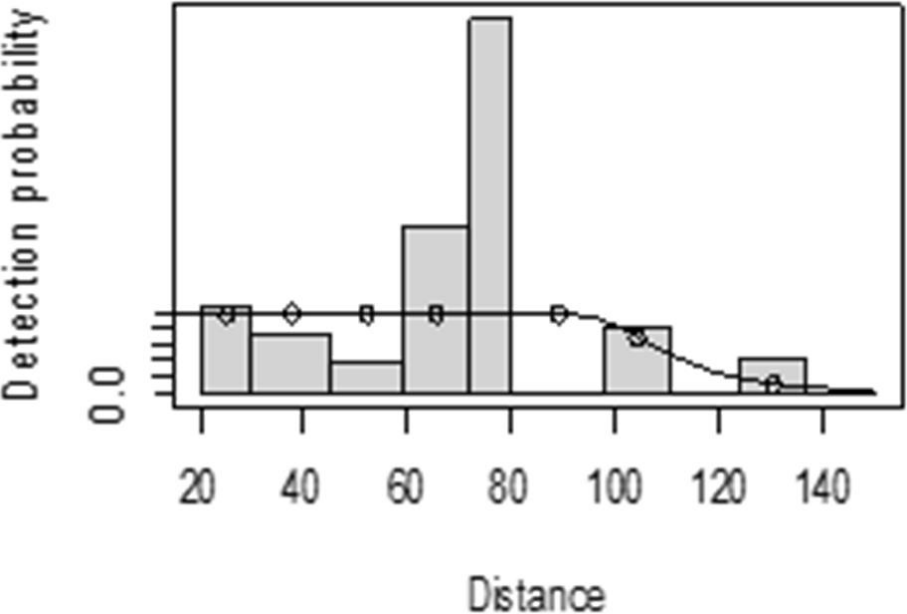
Grant’s gazelle detection function plot during wet season.

For both seasons, all observations beyond the 150 m perpendicular distance were discarded, which is the right truncation. Although individuals could still be observed well above 150 m in open habitats such as grasslands, identifications of individuals by age and sex classes were not apparent. As shown in Fig. 3, excessive detection occurs at 45 to 60 m and 85 to 105 m transect distances. For the wet season, the graph shows that there was no detection at 0 to 15 m perpendicular distance and less detection between 60 to 75 m and 90 m to 105 m (Fig. 4). For Grant’s gazelle in the dry season, the detection function during the dry season showed very low close to the transect and increased with an increase in distance and reaching its highest at a distance 80–100 m, while it recorded fewer and fewer observations as the distance gets longer at 150 m (Fig. 3). For the wet season, there were few observations beyond the 60 m to 80 m interval, which is a high observation (Fig. 4).

**Figure 5:**
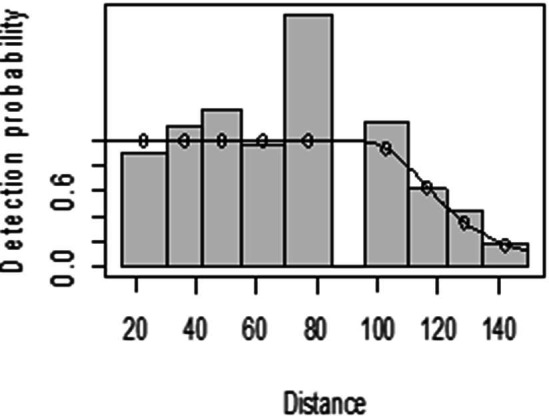
Lesser kudu detection function plot during dry season.

**Figure 6:**
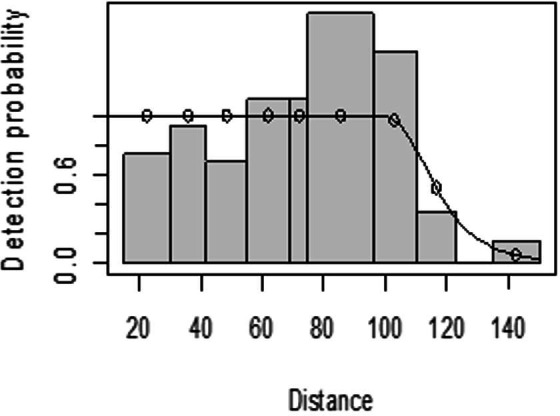
Lesser kudu detection function plot during wet season.

The detection function for kudu in the dry season shows that there was heaping at the interval 69 to 85 m and little detection at the interval 85 to 96 m perpendicular distance. However, most distance interval observations are close to the detection function curve (Fig. 5). In the wet season, there was more discrepancy at the interval 75 to 96 m and 96 to 110 m perpendicular distances (Fig. 6). The irregularity in the detection function curve was due to some topographic and vegetation dispersion irregularities. In some of the cases, valleys and hills and bush tickets were limiting detections even at the nearest detection distances.

### Population structure

#### Age structure

**Figure 7:**
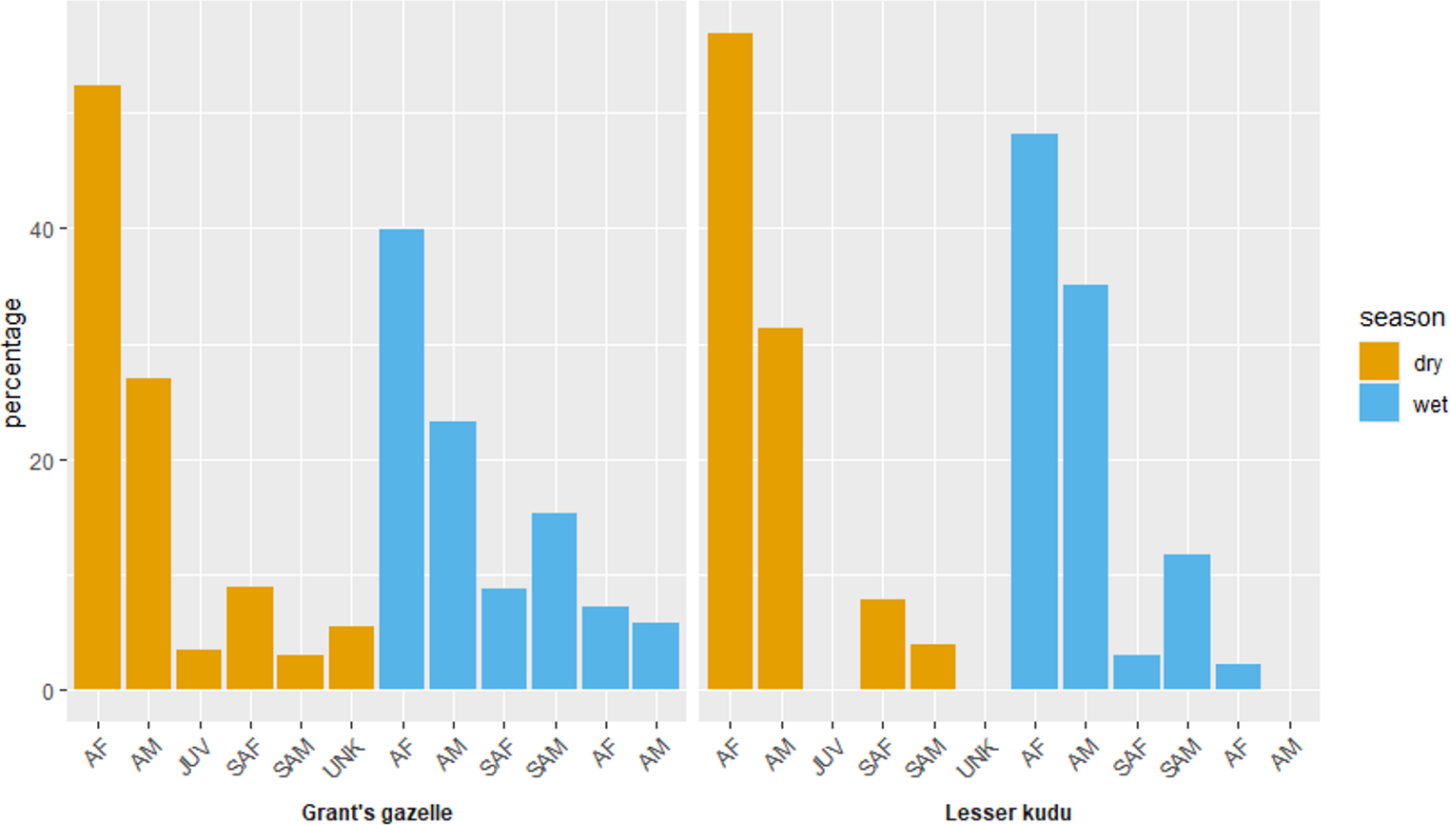
Age and sex ratio of Grant’s gazelle and lesser kudu during both dry and wet seasons. AM, Adult male; AF, Adult female; SAM, Subadult male; SAF, Subadult female; JUV, Juvenile; UNK, Unidentfied.

Adult female comprised the highest proportions for both species during both dry and wet seasons (Fig. 5). Adult female comprised the highest female proportion for Grant’s gazelle during both dry (49.60%) and wet (41.04%) seasons. On the other hand, sub adult male comprised the least percent proportion during both dry (2.40%) and wet (7.46%) seasons (Fig. 7). Adult female comprised the highest female proportion of lesser kudu population during both dry (47.01%) and wet (52.00%) seasons and there was no record of young individuals during dry season (Fig. 7).

#### Sex composition

Grant’s gazelle species comprised 73 individuals of female and 42 individuals of male during the wet season, whereas the number of male individuals decline to 32 individuals and the number of female individuals remained constant (Table 4). Adult females comprised the highest number of individuals of Grant’s gazelle during both dry (62) and wet seasons (55), whereas sub adult female comprised the least number of individuals during both dry (three) and wet (10) seasons (Table 4). Likewise, adult female individuals comprised the highest number of individuals of lesser kudu during both dry (52) and wet (63) seasons and no young individuals of lesser kudu were recorded during dry season (Table 4). Main effect model selected (the interactions between season and age and sex groups), as the dispersion parameter perfectly fitted taken to 1.0, using estimated marginal means (Least-squares means) of Tukey multiple comparison test, young *vs* sub- adult male showed insignificance (*P*-value = 0.122), others showed significance difference (*P*-value = 0). Main effect model selected; dispersion parameter perfectly fitted to 1.0. Tukey multiple comparison test all sexes (female, male, unknown) showed significance differences (*P*-value = 0).

#### Sex ratio

The average adult male to adult female sex ratio (AM:AF) for Grant’s gazelle was (1.0:1.9), whereas the average adult female to young ratio is (1:0.06) (Table 4). The adult male to adult female sex ratio (AM:AF) for the lesser kudu is (1.0:0.03) (Table 4). The sex ratio is skewed towards females.

## Discussion

Accurate and precise estimation of population size and density is critical for applied ecology research, as well as wildlife management and monitoring (Byrne, Parnell & Keeffe, 2021). A reliable estimate of population density for medium and large mammal species is required to develop population dynamics and distribution models, as well as to carry out conservation and management programs (Santini et al., 2022). Furthermore, accurate and unbiased estimation of population is a perquisite for better management of the species (Jacquier et al., 2021). According to the International Union for Conservation of Nature (IUCN) criteria, population size is an important criterion for determining the appropriate level of threat to a wild species (IUCN/SSC-ASG, 2008). The population size estimate of the two species from remote unexplored areas obtained in this study is crucial input for the global population estimate of the two species, to determine appropriate level of threat by the IUCN and ultimately for sustainable wildlife management.

The density of Grant’s gazelle in grassland habitat is comparable with the record in Nechisar National Park (NNP) (Alemu, Mundanthurai & Bekele, 2016). On the other hand, the overall density estimate (1.7 Grant’s gazelle km^−2^) is comparable with Kiffner et al. (2020) (1.5 Grant’s gazelle km^−2^). The density estimates revealed healthy population of the two species at the southeastern extreme border of Ethiopia. The population structure of the two studied species indicates healthy sex and age ratio signifying the prospects of health population growth in the future. This indicates the significance of the national park as important conservation area for Grant’s gazelle. Furthermore, it contributes towards accurate global population estimate and sustainable conservation efforts.

More individuals of Grant’s gazelle were recorded during wet season than dry season. The most probable reason for this might be attributed to human activity and grazing intensity differences between dry and wet seasons as revealed by a previous study in the national park. Due to arid nature of GNP, during the dry season food and water availability declines, implying the probability of individuals of Grant’s gazelle to move out of the national park in search of better food and water sources (Asefa, Mengesha & Aychew, 2017). The findings of the study is consistent with Admasu, Bekele & Asefa (2016) and Alemu, Mundanthurai & Bekele (2016) that stated abundance of species is affected by the availability of food and cover, which is influenced mainly by vegetation composition and structure.

Higher abundance of Grant’s gazelle was recorded in the grassland habitat during the wet season than during the dry season. Grant’s gazelles exhibit an adaptive strategy to survive in changing environmental conditions. They graze on the abundant grass during the wet season. However, as the season shifts to dry and grass becomes scarce, they adapt by switching to browsing for alternative food sources (Kilonzo, Ekaya & Kinuthia, 2005). A study was conducted in Nech Sar National Park, which reported that most Grant’s gazelles vacate the grasslands when there is no rain. They then migrate to bushland and woodlands habitats in search of alternative food sources (Mengesha & Bekele, 2008; Alemu, Mundanthurai & Bekele, 2016).

**Table 4:** Sex ratio of Grant’s gazelle and lesser kudu species during dry and wet seasons.

Species	Season	Sex and age categories					Ratio		
							Sex and age		
		AM	AF	SAM	SAF	YG	AM:AF	AF:YG	AM:YG
Grant’s gazelle	Wet	32	55	10	18	20	1:1.7	1:0.4	01:0.6
	Dry	29	62	3	11	21	1:2.1	1:0.36	01:0.72
	Ava.	30	58	7	14	21	1:1.9	1:0.36	01:0.7
Lesser kudu	Wet	48	63	3	16	4	1:1.3	1:0.06	01:0.8
	Dry	36	52	4	8	0	1:1.8	1: 0.0	01:0.00
	Ava.	42	58	4	12	2	1:1.5	1:0.03	01:0.05

**Notes.**

NB AF Adult Female AM Adult Male SAF Sub adult Female SAM Sub Adult Male YG Young

Gazelles remain within their original ranges or move locally within short distances, where there is enough fodder (Dobson et al., 2006; Herrera-Sánchez et al., 2020). Gazelles congregate to defend themselves from predators and to satisfy their nutritional requirements in areas where resources are plenty as stated by Gebremedhin & Yirga (2006) and Henley, Ward & Schmidt (2007). They feed on herbs and shrubs during the wet and dry seasons, respectively (Mamo et al., 2019). They migrate to other areas in search of water and fodder during dry season, remain within their original habitat or move locally within short distances, where there is enough fodder (Gebremedhin & Yirga, 2006).

The difference in the number of individuals recorded among season and habitat of gazelle in the area could be attributed to seasonal changes in quality and quantity of foraging resources (Mamo et al., 2019; Zhang et al., 2024). The occurrence of higher density of gazelle on the grassland habitat during wet season could be due to better availability of resources in this habitat type during the wet season, fresh grass shoots with rich nitrogen content are available (Mamo et al., 2019; Zhang et al., 2024). During dry season when foods are scare, gazelles also move to the surrounding shrubs and bushes to get enough food that grow outside their original habitats (Henley, Ward & Schmidt, 2007). They disperse into smaller family units and distribute to the surrounding habitats to satisfy their nutritional requirements. The wet season is the most favorable time for Grant’s gazelle population. During this time, the plains also possess high fodder quality and moderate atmospheric temperature for the gazelles to forage.

The population of lesser kudu recorded in GNP is comparable with populations recorded in other protected areas. For example, as compared to the study in Tululujia Wildlife Reserve (573 individuals) in southern Ethiopia, the population estimate of lesser kudu is double in GNP (1,008 individuals). The study revealed healthy population of the species at southeastern extreme boarder of Ethiopia. The age and sex structure indicated a population that has growing future prospect. This indicates the significance of the national park as important conservation area for Grant’s gazelle. Furthermore, it contributes towards accurate global population estimate of the species.

Generally, higher individuals of lesser kudu were recorded in GNP during the wet season than dry season. Lesser kudus were more abundant in the bushland habitat (570 and 395) and had a density of *D* = 3.43 ± 1.38 and 2.37 ± 0.88 in wet and dry season, respectively. The possible reason for the decrease in population during the dry season compared to the wet season might be due to availability of resources (food and water) and minimum competition and the type of habitat selection by vegetation type among habitat and between seasons might affect it as stated by Waltert, Meyer & Kiffner (2009). According to Estes (2012), adult male lesser kudu has beautiful shaped horns, used as musical instruments, honey containers and symbolic ritual objects in many places. In some cultures, the horns are thought as the dwelling places of powerful spirits, and symbols for male potency/strength (Kingdon, 2015). Consequently, adult male lesser kudu are one of the best targets of most trophy hunters, leading to their lower numbers compared to other age categories in many places (Crosmary, Côté & Fritz, 2015). Therefore, poaching could be more prevalent during dry season, since during dry seasons most herbaceous plant communities dry and some trees and shrubs shade their leaves creating more open habitat (Matsuo et al., 2024).

Moreover, the difference in availability of resources between dry and wet seasons could create population abundance difference between seasons. During dry season, due to limitations in rainfall availability the vegetation cover becomes sparser and food and water availability becomes limited (Matsuo et al., 2024; Waltert, Meyer & Kiffner, 2009). Lesser kudus due to their shy behavior are rarely found in open habitat and flat and thicketed bush and woodland habitats. Leuthold (1971) states that the lesser kudu a forest-dwelling antelope, exhibits vigilant or shy behaviors. This behavior can be attributed to its feeding habits as a pure browser, consuming foliage from bushes and trees, as well as fruits, seeds, and herbs. The frequency of fire and relatively high habitat disturbance during the dry season may be the reason for the lower numbers of lesser kudus in the study area during this season (Gebo, Takele & Shibru, 2023).

Lesser kudus were observed in woodland, bushland and wooded grassland of GNP. This might be due to the availability of more browsing in woodland and bushland plains. This could be related to its ability to eat a greater variety of woody plant species that provide browse and cover, as well as its adaptation to a particular area that protects it from predators (Vaughan, Ryan & Czaplewsky, 2000; Gray et al., 2007). This finding agrees with the findings of Belete & Melese (2016) that reported the lesser kudu was observed in all habitats such as woodland, bushland and wooded grassland, due to its wide habitat range and tolerance abilities, the kudu may use a variety of plant species as alternative food. Similarly, Belete & Melese (2016) reported that the forested area is a preferable habitat for lesser kudu since it desires dense cover to protect itself from any dangers. The lesser kudu exhibits wary behavior and has a wide habitat range. Lesser kudu is a browser or intermediate feeder that inhabits dense woodlands (Leuthold, 1971). Generally, bushland, wooded grassland, and grassland habitat supported more population as compared to woodland, this might be due to the habitat preference characteristics of the species.

Understanding the principle dynamics of animal populations is important in wildlife management. The sex ratio in a population is an important factor in population growth (Vetter & Arnold, 2018; Tarsi & Tuff, 2012). An imbalance often leads to a poor mating frequency (Tomillo, 2022). Therefore, sex ratios should be monitored on an ongoing basis. The results of the study revealed important information on age and sex structure and sex rations of the two ungulate species under study. Hence, the information is crucial for determining the future population prospect of the two species.

Sub-adult females comprised the highest proportion of age and sex category for both Grant’s gazelle and lesser kudu during both dry and wet season. This indicates the potential of the populations of the two species to increase in the future under favorable condtions (Vetter & Arnold, 2018). As compared to adult females the number of individuals of adult male recorded was lowest during both wet and dry seasons. Competition of males also forces the bachelor males to migrate to less suitable habitats that are poor in food quality, and exposing them to predators and hunters. Adult males are solitary, fighting and competition for food and mating might possibly force bachelor male to marginal habitat that are exposed to predators’ attack. Similar results were reported on Grant’s gazelle in National Park (Alemu, Mundanthurai & Bekele, 2016). Alemu, Mundanthurai & Bekele (2016), explained that possible reason for an unequal sex ratio and less proportion of adult male in most of the species might be related to poaching pressure in which the adult males are mostly selected by poachers and predation pressure on males or emigration of male to other habitats for food. Adult males are mostly poached because of the relatively large volume of bush meat, horn, and skin they provide, and also hunting adult male is a sign of bravery in most indigenous African cultures (Angula et al., 2018). Male-biased hunting practices of the species by local communities might contribute to the smaller number of adult males as stated by (Mamo, Asefa & Mengesha, 2015; Ritchot et al., 2021). Similar results reported by Belete & Melese (2016) in Tululujia Wildlife Reserve (TWR) (Alemu, Mundanthurai & Bekele, 2016) in NNP. Competition of males could also force the bachelor males to migrate to less suitable habitats that are poor in food quality and exposing them to predators and hunters (Alemu, Mundanthurai & Bekele, 2016).

Comparatively lower proportion of young are observed during the dry season. The higher number of young recorded during the wet season indicates that births happen primarily in the wet season. The habitat may not be suitable for juveniles during the dry season; particularly because the juveniles are susceptible to the predators. Observation of low juveniles revealed, there might be mortality due to high level of predation, habitat change, and long drought factors. The difference in number of young between dry and wet season might be due to females giving birth to juveniles during the wet season. Young are usually hidden inside the dense and tall grasses and in the bushes during the dry season, until they are strong enough to run fast and escape from predators.

This might be because young are more vulnerable to predators and hidden under the grasses and vegetation to escape from predators (Grovenburg et al., 2012). The dry season and droughts might cause more mortality than other times leading to age-sex dependent mortality where most youngs and olds are killed. A female-biased sex ratio has been reported in many antelope populations. Potential bias in sex ratio is related with the management of the species (Alemu, Mundanthurai & Bekele, 2016; Gebo, Takele & Shibru, 2023). A female-biased sex ratio increased reproductive success in the future prospect of the population (Ritchot et al., 2021).

## Conclusions

The study indicated that GNP is home to healthy populations of Grant’s gazelle and lesser kudu. The population estimate for the two species is crucial to an accurate global population estimate of the species. Seasonal variation in abundance of the two study species has been exhibited due to seasonal variation in habitat components such as food, cover and water. Generally, the population structure was skewed towards females and a considerable young population indicating good future population prospect for both Grant’s gazelle and lesser kudu. The study is critical for baseline information for population monitoring, population regulation by National Park managers and other researchers. The study has provided valuable data on the current population size and population structure of Grant’s gazelle and lesser kudu which will be used as baseline data for future monitoring data to compare the population changes, examine the causes of the changes, and take management measures. Therefore, to promote sustainable management of the species there is a need for periodic population monitoring and enhanced conservation efforts.

##  Supplemental Information

10.7717/peerj.18340/supp-1 Supplemental Information 1 Raw data

## Additional Information and Declarations

### Data Availability

The following information was supplied regarding data availability: The raw data that supports the findings of the study is available in the Supplemental File.
